# Junctional ectopic tachycardia in infants and children

**DOI:** 10.1002/joa3.12282

**Published:** 2019-12-03

**Authors:** Ranjit I. Kylat, Ricardo A. Samson

**Affiliations:** ^1^ Department of Pediatrics College of Medicine University of Arizona Tucson AZ USA; ^2^ Children's Heart Center of Nevada Las Vegas NV USA; ^3^ Department of Pediatrics Division of Cardiology University of Nevada‐Las Vegas School of Medicine Las Vegas NV USA

**Keywords:** amiodarone, cardiomyopathy, catheter ablation, dilated, ectopic junctional, electrophysiology, tachycardia

## Abstract

Tachyarrhythmias originating in the atrioventricular (AV) node and AV junction including the bundle of His complex (BH) are called junctional tachycardia (JT) or junctional ectopic tachycardia (JET). Congenital JET (CJET) is a rare arrhythmia that occurs in patients without a preceding cardiac surgery and can be refractory to medical therapy and associated with high morbidity and mortality. CJET has a high rate of morbidity and mortality with death occurring in 35% of cases. JET occurring within 72 hours after cardiac surgery is referred to as postoperative JET (POJET) and caused by direct trauma, ischemic, or stretch injury to the AV conduction tissues during surgical repair of congenital heart defects. Focal junctional tachycardia (FJT) is also known as automatic junctional tachycardia and includes paroxysmal or non‐paroxysmal forms. We discuss a staged approach to therapy with improved pharmacological therapies and the use of catheter‐based therapies.

## Graphical abstract

A critical review of junctional ectopic tachycardia in children including congenital, postoperative, and focal paroxysmal and non‐paroxysmal forms.

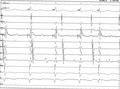

**Abbreviations**
AV: Atrioventricular
AVNRT: AV nodal reentrant tachycardia
AVRT: AV reciprocating tachycardia
EKG: electrocardiogram
SVT: Supraventricular tachycardia
TIC: tachycardia‐induced cardiomyopathy

## 1 INTRODUCTION

**Figure 1:**
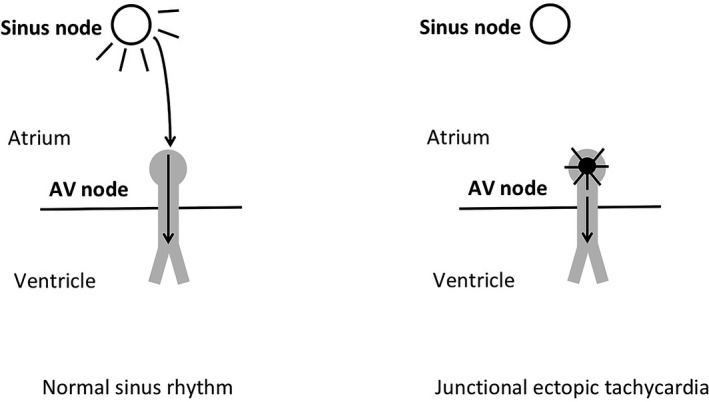
Schematic of mechanism of junctional ectopic tachycardia. Right: In normal sinus rhythm, spontaneous depolarization of the sinus node results in propagation of electrical signal through the atrium. Conduction of the electrical signal occurs through the AV node to the ventricle via the bundle branches and His‐Purkinje system. Left: In junctional ectopic tachycardia, spontaneous depolarization occurs in the AV node (ie, junction) and conducts to the ventricle. There may or may not be conduction of the signal retrograde into the atrium

Tachyarrhythmias originating in the atrioventricular (AV) node and AV junction including the bundle of His complex (BH) are called junctional tachycardia (JT) or junctional ectopic tachycardia (JET).^1^, ^2^, ^3^ AV junction refers to the AV‐specialized conducting system consisting of the transitional cell zone, the AV node and its extensions, and the penetrating part of the BH. It has also been called His bundle tachycardia and junctional automatic tachycardia. JET is generally rapid with narrow QRS complex but can be regular or irregular.^1^, ^2^ It could have retrograde atrial conduction in a 1:1 pattern or AV dissociation with variable conduction to the atria. JET does not involve a reentry circuit. (Figure 1) Thus, reentrant tachycardias, which require part of the AV node as part of their circuit, such as AV reentrant tachycardia (AVRT) and AV nodal reentrant tachycardia (AVNRT), are not classified as JT.^1^, ^2^ The types of JET based on their etiology are congenital (CJET), postoperative (POJET), and focal paroxysmal and non‐paroxysmal forms and those associated with digitalis toxicity.^1^, ^2^ In infants and children, the focal and congenital variety are not as common as postoperative which is seen in up to 5% after cardiac surgery.^3^

## 2 CONGENITAL JUNCTIONAL ECTOPIC TACHYCARDIA

CJET is a rare arrhythmia that occurs in patients without previous cardiac surgery. This is often refractory to medical therapy and associated with high morbidity and mortality.^4^, ^5^ CJET was initially described by Coumel.^6^ It has also been called familial JET, chronic idiopathic JET, and primary JET. It occurs in the first 6 months of life and is usually incessant.^4^, ^5^, ^6^ Data from major electrophysiology (EP) centers over four decades identified only less than 100 patients.^1^, ^2^, ^5^

### 2.1 Etiopathophysiology and mechanism

A mechanism of enhanced automaticity has been postulated.^1^, ^2^, ^7^, ^8^ This could explain why it is unresponsive to adenosine and DC cardioversion emphasizing the automatic (rather than reentrant) nature of this tachycardia. In addition, the irregular tachycardia rate, the lack of dependence on a critical coupling interval for tachycardia initiation, and the inability to entrain the arrhythmia also suggest a focal automatic (ie, non‐reentrant) mechanism.^8^ Other mechanisms that may be responsible are abnormal automaticity and triggered activity originating from the BH.^1^, ^8^ The difference between enhanced automaticity, is that abnormal automaticity does not demonstrate overdrive suppression because of its reduced resting membrane potential of less than −60 mV. The sodium current and electrogenic pump are in an inactivated state and the abnormal automaticity is dependent on the slow inward calcium current.^8^ This would explain why it is sensitive to calcium channel blockers.^8^ Adenosine does not have any effect on abnormal automaticity, but it can transiently suppress enhanced automaticity. Therefore, sensitivity of an automatic tachycardia to calcium channel blockade and insensitivity to adenosine are consistent with this abnormal automaticity.^8^ In a subset of cases which can be terminated with adenosine, another mechanism postulated is triggered activity where the arrhythmia can be induced and terminated with programmed stimulation.^8^

Multiple gene deletions like the angiotensin‐converting enzyme insertion/deletion (ACE I/D) and troponin I‐interacting kinase (TNNI3K) have been implicated to predispose to CJET and POJET.^9^, ^10^, ^11^ TNNI3K is a cardiac‐specific gene, encoding a cardiac troponin I‐interacting kinase. Mutations here or in its gatekeeper could lead to altered phosphorylation of its substrates, such as cardiac troponin I, and may result in cardiac dysfunction, cardiac arrhythmia, and dilated cardiomyopathy.^11^, ^12^ Speculation of the familial basis is based on theories that calcium dysregulation or channelopathies may be a cause and is based on finding this mutation in families, presence of a Brugada electrocardiogram (EKG) pattern in CJET, and with the association of sudden death.^11^, ^12^, ^13^ In some cases where the etiology was not certain, there are reports that it could be related to viral myocarditis.^14^, ^15^ There is an association with other congenital heart defects like ventricular and atrial septal defects. Histologic abnormalities like inflammation, fibrosis, focal degeneration, and fibroelastosis involving the AV node region and BH, division of AV node, left‐sided AV node, entrapment, and distortion have been reported.^1^ A family history with similar arrhythmia was seen in up to 50% of all cases.^1^, ^7^, ^8^

### 2.2 Clinical presentation and natural history

The presentation is usually early in infancy and generally within 6 months but the diagnosis is often delayed. Presentation as a fetal tachycardia prenatally has been reported in up to a third of the CJET patients.^1^, ^16^ This could be associated with symptoms of congestive heart failure (CHF) or as hydrops in the fetus. During the tachycardia, heart rate typically ranges from 200 to 250 per minute.^8^ At the time of diagnosis, patients may present with cardiomegaly, cardiac failure, dilated cardiomyopathy, and occasionally ventricular fibrillation.^3^, ^4^ CJET can lead to third‐degree (complete) block and sudden cardiac death.^4^, ^5^, ^17^

Collins et al, reviewed 94 patients with non‐POJET.^18^ In this series, age at presentation was equally divided between patients younger than 6 months and older than 6 months. Unlike earlier reports, a familial association was noted in only 20% of patients. The younger infants had a faster and incessant tachycardia compared to children older than 6 months, who had slower and sporadic tachycardia.^18^ Villain et al, reported 26 infants less than 6 months of age with CJET and found 61% had CHF.^7^ About 35% of these patients died. There was a positive family history in 50% of these patients.^7^ The incessant nature of the tachycardia may lead to secondary tachycardia‐induced cardiomyopathy (TIC) and CHF.^7^

Infants with CJET have incessant tachycardia and faster rates resulting in poor systolic function and ventricular dilatation leading to a higher likelihood of CHF. Some of these infants may initially appear compensated only to subsequently develop cardiovascular collapse. The cause of death in these patients is typically sudden without clear etiology, but development of paroxysmal third‐degree heart block has been documented.^1^, ^7^, ^17^, ^19^ The clinical characteristics are a gradual onset, or "warming up" phase, and a gradual termination, or "cooling down" phase and rate variability.^19^, ^20^ Those patients who do not succumb to early hemodynamic compromise will show spontaneous slowing of the junctional rate over time. However, subsequent Holter evaluation many years after symptom resolution may show persistent JET.^9^ Causes of death include ventricular fibrillation, AV block, and refractory heart failure. In more recent reports of patients with CJET, mortality was 4%–9%.^9^, ^18^

### 2.3 EKG

**Figure 2:**
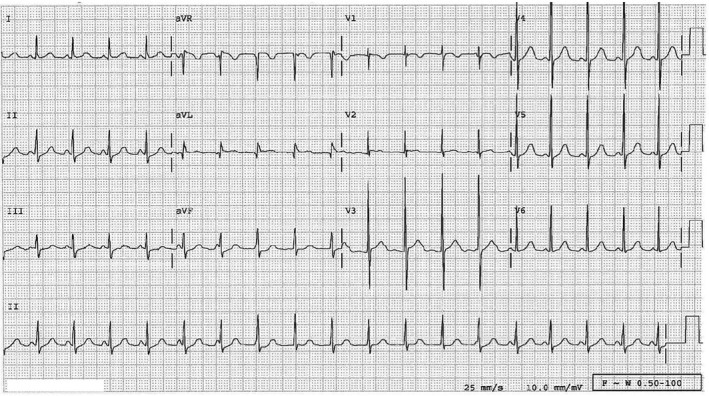
12‐lead ECG of a 7‐year‐old with congenital junctional tachycardia. The patient is on chronic medications including propranolol, digoxin, and flecainide. Note the narrow QRS complexes indicative of conduction within the ventricle through the His‐Purkinje system. Also note AV dissociation with sinus P waves “marching through” the rhythm

The QRS is usually normal and narrow complex, and is identical to a normally conducted sinus beat. There is often complete ventriculoatrial (VA) dissociation. Retrograde P waves may be seen in the terminal portion of the QRS. The atrial rate is exceeded by the rapid junctional rate and the slower sinus dissociated P waves may be seen. (Figure 2) However, AV dissociation may not always be seen, as passive VA conduction can sometimes occur over the AV node in a pattern of retrograde Wenckebach or, less commonly, a 1:1 pattern.^7^, ^8^, ^20^ If the diagnosis is obscured by passive retrograde conduction, administration of adenosine will result in complete VA dissociation without altering the rapid junctional rate.^20^ When the diagnosis is unclear from a surface EKG, esophageal EP testing can be helpful to discern atrial activity.^21^ The diagnosis of JET can be confirmed by VA disassociation during JET noted with either ambulatory monitoring or during the EP study, and reinforced with the typical rate variability associated.^22^ Occasionally, the tachycardia might be irregular, thus resembling atrial fibrillation. Rates for JET can vary over time in response to adrenergic state, and occasional irregularity may be seen owing to capture beats from dissociated sinus rhythm.^20^

### 2.4 Electrophysiology

**Figure 3:**
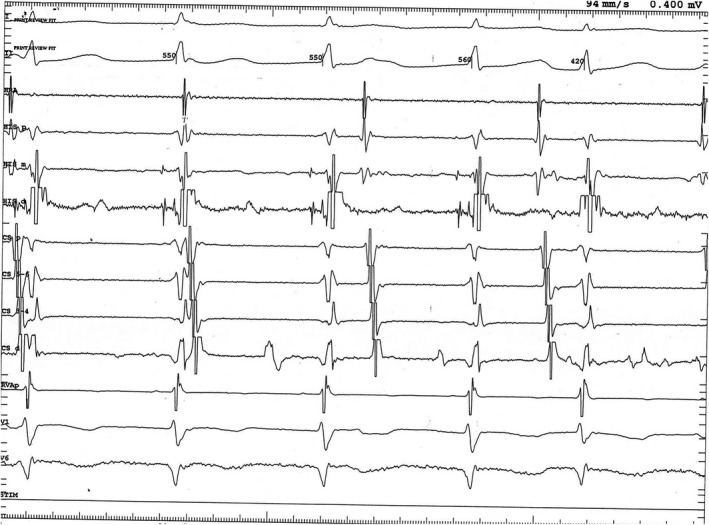
Intracardiac recording of junctional ectopic tachycardia in the same 7‐year‐old patient as in Figure 2. Note the earliest deflection in the His bundle catheter “mid” electrodes (HIS m), indicating origin of the tachycardia in this region. HRA, high right atrium; HIS p, proximal His bundle catheter electrodes; HIS d, distal His bundle catheter electrodes; CS p, proximal coronary sinus catheter electrodes; CS 5‐6, second most proximal coronary sinus catheter electrodes; CS 3‐4, second most distal coronary sinus catheter electrodes, CS d, distal coronary sinus catheter electrodes

During EP study of the patient in sinus rhythm, there is a normal His‐ventricle (HV) interval and normal AV conduction curves. In some cases, tachycardia can be induced by atrial ectopics or rapid atrial or ventricular pacing, suggesting abnormal automaticity or triggered activity as the possible mechanism. Enhanced automaticity can also occur, however, as suggested by the ability of this tachycardia in some patients to accelerate with enhanced sympathetic tone.^2^, ^22^, ^23^, ^24^, ^25^ The arrhythmia usually is not inducible by a single extrastimulus, cardioversion, or by programmed electrical stimulation, thus making reentry an unlikely mechanism. (Figure 3).

As a consequence of the incessant nature, the patient is more likely to be seen in the EP laboratory in tachycardia. Pacing maneuvers can be considered at a similar cycle length to the tachycardia. Characteristics of JET include the lack of resetting or termination of the tachycardia during His‐refractory atrial extrastimulus pacing or a His‐atrial time which is longer with ventricular pacing than during the arrhythmias.^2^, ^22^, ^24^ If an atrial extrastimulus is delivered during tachycardia and advances the timing of the His immediately following without terminating the tachycardia, this indicates that a retrograde fast pathway is not required for the maintenance of the tachycardia, thus confirming the mechanism as JET.^24^ Overdrive pacing during tachycardia can be used to differentiate from reentrant arrhythmia. Upon cessation, the atrial‐His‐His‐atrial response in JET would reveal re‐initiation of the JET post‐pacing with the His signal as the onset of tachyarrhythmia.^2^, ^22^, ^25^ Administration of adenosine results in VA dissociation without termination.

### 2.5 Management

Medical management is the mainstay of initial treatment for most patients but adequate control of the tachycardia is often elusive, with patients typically requiring two or more antiarrhythmics.^8^, ^18^, ^26^ Administration of adenosine will result in VA dissociation without termination of the dysrhythmia. Amiodarone is the initial treatment of choice and is used most frequently, as a first‐line agent and has been used either alone or in combination with propranolol or flecainide in infants. Caution must be exercised when administering amiodarone intravenously because of the frequent occurrence of hypotension.^27^ The addition of a second drug may help reduce the dose of amiodarone and its potential long‐term toxicity.^1^, ^26^ Digoxin is not effective and there are reports of it precipitating heart failure, worsening tachycardia, or causing ventricular fibrillation.^8^ Successful use of regimens of flecainide with propranolol has also been reported.^28^ Ivabradine, which works by selective inhibition of hyperpolarization‐activated cyclic nucleotide‐gated channels, has been shown to be effective.^29^, ^30^, ^31^ It has been shown that selective blockade of the rapidly activating delayed rectifier K+channel (I(Kr)) by pure class III antiarrhythmic drugs like nifekalant has been useful in CJET refractory to amiodarone and other drugs.^32^ Its mechanism of action is unique in that it does not affect the calcium or sodium channel or the inward rectifier potassium current.^32^ With early pharmacologic management, partial efficacy is seen in 70% of cases, while only 10% of patients have complete suppression. The remaining 20% of cases are completely resistant to medical therapy.^8^ The use of multiple antiarrhythmic drugs adds to the risk of sudden death attributable to its potential pro‐arrhythmic effects.^8^

### 2.6 EP and catheter ablation

Prior to the introduction of catheter ablation, surgical dissection and cautery of the AV junction were used for refractory CJET with the invariable sequelae of permanent AV block. Ablation was introduced with the hope that it could suppress the tachycardia while preserving AV conduction. Because of the proximity of the normal conduction system to the JET focus, the initial challenges were of precisely mapping the JET focus by EP. Early on, ablation carried a significant risk for a permanent third‐degree AV block and the need for permanent pacing.^8^ Refinement of the techniques of ablation over the last two decades has led to a higher rate of normal intracardiac conduction, but careful selection of patients is needed, as complications are high, especially in infancy.^1^, ^8^, ^22^

During JET episodes, if VA conduction is present, the site of earliest atrial activation can be mapped and targeted for ablation.^8^ If VA conduction is not present during JET, empiric lesions can be applied sequentially in the posterior septum (the slow AV nodal pathway region), midseptum, and then the anterior septum. This is associated with a much higher risk for permanent AV block.^8^ When the BH electrogram is difficult to obtain, para‐Hisian pacing helps to determine the proximity to the His bundle, so that ablation can be avoided in the vicinity to decrease the risk of complete heart block. Suppression of JET during application of energy usually suggests success. Radiofrequency ablation had been used extensively for JET including in infants.^1^, ^2^, ^8^, ^33^, ^34^ Radio‐frequency (RF) ablation in children less than 2 years was successful in only 74% when compared to 91% in children over 2 years.^34^

Three‐dimensional (3‐D) mapping systems and atrial overdrive pacing improve the assessment of AV nodal function and are now used routinely prior to ablation.^8^, ^22^, ^23^ Cryothermal JET ablation has the advantage that it has less risk of producing a permanent third‐degree AV block.^18^, ^20^, ^22^, ^35^, ^36^, ^37^ One advantage of cryoablation is its ability to ablate during the mapping process at −30°C (−22°F), and this ablative injury is reversible. Once tachycardia suppression is confirmed and the presence of AV block assessed, then full ablation at −70°C (−94°F) can be embarked upon and this causes more permanent cryothermal injury.^8^, ^22^

Refinements in electrophysiological mapping techniques further help to localize targets.^8^, ^22^, ^33^, ^37^ The mapping catheter is moved along the His bundle from proximal to distal. At more proximal His bundle sites (where ablation is likely to be unsuccessful and may cause transient AV block), the HV interval is shorter during ectopy than during sinus rhythm, and the local unipolar His bundle recording shows an “RS” morphology. Once the mapping catheter reaches a more distal successful ablation site, the HV interval during ectopy is identical to the HV interval during sinus rhythm, and unipolar recording of the His bundle electrogram shows a “QS” morphology during ectopy. During 3D mapping, geometry of the right atrium and triangle of Koch (TOK) are obtained and the sites of His potentials and coronary sinus os are anatomically tagged. If there are spontaneous runs of JET, test applications of cryothermal energy can be delivered. If there is no VA dissociation, preablation testing can distinguish JET from AVNRT by attempting to separate the atrial and the ventricular signals during the arrhythmia (with premature atrial beat introduction, overdrive pacing, or adenosine). Electrograms help to confirm catheter location within the TOK.^22^ As JET does not display electrical signals to allow targeted ablation of the ectopic focus, anatomic landmarks, the AV ratio, and the His electrogram in addition to 3D mapping can be utilized to assist with catheter localization within the TOK. Patients who do not have spontaneous runs of JET, isoproterenol administration, or pacing maneuvers can be attempted to induce the arrhythmia. However, if VA conduction is not present, the procedural approach for these patients is much like an anatomic approach for slow pathway ablation, ablating at the earliest site of atrial activation when patients exhibit 1:1 VA conduction but may not correlate to the site of successful ablation.^22^ Instead of an electrically guided ablation, it has been suggested to use an anatomic‐based approach applying energy starting in the posteroseptal region and moving anteriorly if the tachycardia persists.^22^ As with slow pathway modification, the location of successful ablation of the JET focus was generally located within the slow pathway area of the TOK.

Overall, both RF and cryoablation have 80%‐85% initial success with recurrence rates of 13%‐14%.^1^, ^8^ Cryoablation has a much lower risk for inadvertent permanent AV block at close to 0%.^8^ The site for successful JET elimination is not the same in all patients; AV block may be a risk when the focus of automaticity seems to be arising from the anteroseptal area rather than more posterior in Koch's triangle.^20^

## 3 POSTOPERATIVE JUNCTIONAL ECTOPIC TACHYCARDIA (POJET)

In children, especially infants, POJET can be seen within the first 72 hours after cardiac surgery.^1^, ^2^ POJET is caused by direct trauma, ischemic, or stretch injury to the AV conduction tissues during surgical repair of congenital heart defects.^1^, ^20^

### 3.1 Incidence and Risk Factors

After the correction of congenital cardiac defects, POJET is seen in 1%‐15% of children.^1^, ^2^, ^20^

The risk factors associated with an increased incidence include the age of infant less than 6 months, the postoperative use of dopamine or milrinone, the use of prolonged aortic cross‐clamp and cardiopulmonary bypass, and total surgical time.^1^, ^2^, ^3^, ^8^, ^38^ The use of nitroprusside was associated with a reduced incidence of JET.^8^ The type of cardiac surgery is also a factor, especially those that involve the crux of the heart including tetralogy of Fallot, AV canal, and ventricular septal defect repair, as well as repair of anomalous pulmonary venous return, arterial switch operation, Norwood procedure, and interrupted aortic arch repair.^1^, ^20^, ^38^ Other risk factors associated are postoperative higher core temperature and imbalances of electrolytes.^39^, ^40^, ^41^, ^42^ Of note, all arrhythmias, including JET, are common in those with complex congenital heart disease especially the heterotaxy syndromes but the difficulty in the risk association is that most of these infants have had early surgical interventions.^43^, ^44^, ^45^ The right atrial isomerism is unique in that the association of paired sinus and AV nodes makes the association of JET likely although less common than SVT.^43^

### 3.2 Etiopathogenesis

Several mechanism have been postulated for POJET, including fluid and electrolyte shifts, trauma, stretch, local edema, or ischemia in the region of the AV node or BH. POJET behaves like an automatic arrhythmia displaying warm‐up and cool‐down transitions to sinus rhythm. The onset of POJET is typically during first 24 hours after cardiac surgery and it is seen more often during the rewarming phase. As a result of simultaneous activation of the atria and the ventricles from impulses originating in the BH, the atria contracts against closed AV valves.^1^, ^2^ Thereby decreased ventricular filling results in reduced cardiac output and hypotension.

### 3.3 Diagnosis

The diagnosis is generally made in the presence of VA dissociation, or dissociation with a 1:1 conducting arrhythmia and those that have consistent retrograde conduction. The VA dissociation could have variable conduction to the atria. But visualization of the onset or offset of the tachycardia either spontaneously or after diagnostic maneuvers such as overdrive pacing or administration of adenosine is necessary for an accurate diagnosis.

### 3.4 Treatment

The most important steps in the management are to attempt to reduce the catecholaminergic stimuli along with hypothermia, if needed.^42^ Hypothermia to 32‐34°C either using extracorporeal cooling or intravenous cooling is effective in many cases.^1^, ^2^ A staged approach is generally chosen.^42^ Atrial pacing at 10‐20 bpm above the JET rate can improve AV synchrony and cardiac output, but the JET rate needs to be below 180 bpm. Digoxin, beta‐blockers, and calcium channel blockers are best avoided. Amiodarone is an effective and widely used antiarrhythmic for the treatment of POJET.^27^ It is administered as a continuous maintenance infusion after a loading dose and it has dose‐dependent success in POJET.^27^ There is a high rate of clinically significant adverse events such as hypotension, bradycardia, and AV block and appear to be dose‐related. Hence, it should be used with caution when treating children with critical arrhythmias.^27^ Magnesium sulfate infusion perioperatively also reduces the risk of POJET.^1^, ^2^ Dexmedetomidine, an alpha‐ 2‐adrenergic receptor agonist, is also very effective.^1^, ^46^, ^47^ Flecainide, procainamide, and propafenone have also been used.^48^, ^49^ More recent reports of medications like sotalol, nifekalant hydrochloride, landiolol hydrochloride are being used successfully with minimal adverse effects.^1^, ^50^, ^51^, ^52^, ^53^ The use of prophylactic dexmedetomidine has been successful in preventing many arrhythmias.^54^ The vast majority of POJET is self‐limited and resolves by 72 hours.

There is a morbidity associated with POJET with an increased duration of mechanical ventilation and hospital stay but the previously reported mortality has been dramatically reduced with current management protocols including rapid weaning of catecholaminergic agents, correction of fever, sedation, and correction of electrolyte disturbance.^1^, ^2^, ^42^ Catheter ablation procedures are usually not needed.

## 4 FOCAL JUNCTIONAL TACHYCARDIA

Focal junctional tachycardia (FJT) is also known as automatic junctional tachycardia and includes paroxysmal or non‐paroxysmal forms.^1^, ^55^, ^56^, ^57^ As opposed to CJET, which involve infants less than 6 months of age, descriptions of FJT often involve older children and adults. The paroxysmal form usually has an acute onset and terminates abruptly, but can recur, although both forms are not incessant. In children, they are often asymptomatic but can have vague symptoms like headache, lightheadedness, palpitations, or syncope. However, a rapid or irregular heart rate on physical exam warrants further evaluation.

The non‐paroxysmal FJT is more common in adults and is also called accelerated AV junctional rhythm, and could be related to acute myocardial ischemia, digoxin toxicity, chronic obstructive pulmonary disease, rheumatic carditis, electrolyte disturbances, or could occur after cardiac surgery.^1^ The rates are relatively slower and hence could have minimal symptoms unless the arrhythmia is prolonged. However, symptoms and hemodynamic instability (syncope) have been described in pediatric and adult patients. The non‐paroxysmal form may persist for decades before diagnosis.

### 4.1 Etiology

The likely mechanism of FJT appears to be one of the enhanced automaticity within the AV node or triggered activity attributable to delayed afterdepolarizations. The clinical tachycardia features (catecholamine sensitivity, warm‐up period) and invasive data (showing antegrade AV block above the level of the His bundle in response to beta‐blocker administration) support this. Some cases of adenosine sensitivity support the mechanism of triggered activity in the AV node region.

The usual electrocardiographic finding is a narrow QRS tachycardia with short RP interval or AV dissociation. Occasionally, the tachycardia might be irregular, thus resembling atrial fibrillation. During tachycardia, there is a normal or increased HV interval with AV dissociation that is interrupted by frequent episodes of VA conduction with earliest atrial activation in the posteroseptal, anteroseptal, or midseptal regions.^55^

### 4.2 Treatment

Treatment is only rarely needed and ambulatory Holter monitoring and exercise stress testing should be performed to assess tachycardia burden during catecholamine stimulation and serial echocardiograms are necessary to monitor ventricular function.^1^ If symptomatic, beta‐ blockers, amiodarone, procainamide, or flecainide may be used for acute therapy, chronic therapy, sotalol, amiodarone, or flecainide, or propafenone can be used.^1^, ^56^ In refractory cases, combination treatment with amiodarone or cryothermal catheter ablation may be needed.

The strategy for ablation is as described above or at the site of earliest atrial activation in patients with VA conduction. Empiric slow pathway ablation in the setting of VA block can also be undertaken.^1^, ^22^, ^57^

## 5 CONCLUSION

Of the different forms of JET, CJET remains a rare and difficult arrhythmia to manage. However, significant morbidity and mortality still exist, even with improved pharmacological therapies and the development of catheter‐based therapies. For POJET, a staged approach to therapy is still recommended. In CJET, medical management is carried out first followed by cryothermal ablation if the patient is critically ill, unstable, or has an undue amount of tachycardia burden.

## CONFLICT OF INTEREST

The authors declare no conflict of interests for this article.

## AUTHOR CONTRIBUTION

Both authors contributed in the conceptual design, writing, revision, and editing.
